# Emotional Availability as a Moderator of Stress for Young Children and Parents in Two Diverse Early Head Start Samples

**DOI:** 10.1007/s11121-021-01307-7

**Published:** 2021-11-13

**Authors:** Neda Senehi, Marjo Flykt, Zeynep Biringen, Mark L. Laudenslager, Sarah Enos Watamura, Brady A. Garrett, Terrence K. Kominsky, Hannah E. Wurster, Michelle Sarche

**Affiliations:** 1 Department of Psychiatry, University of Colorado Anschutz Medical Campus, 31001 E 17th Place, Aurora, CO USA; 2 Faculty of Social Sciences/Psychology, Tampere University, Tampere, Finland; 3 Faculty of Medicine/Psychology, University of Helsinki, Helsinki, Finland; 4 Department of Human Development and Family Services, Colorado State University, Fort Collins, USA; 5 Department of Psychology, University of Denver, Denver, USA; 6 Cherokee Nation Behavioral Health, Tahlequah, OK USA; 7 Colorado School of Public Health, University of Colorado Anschutz Medical Campus, Aurora, USA

**Keywords:** Parental adverse childhood experiences, Emotional availability, Hair cortisol, Intergenerational transmission of adversity, Buffering

## Abstract

Positive parent–child relationship quality is critical for buffering children from the effects of stress on development. It is thus vital to develop interventions that target parent–child relationship quality for families experiencing stress. We examined the moderating role of parent–child relationship quality (as measured by parental emotional availability [EA]) in the intergenerational association between parental adverse childhood experiences (ACEs) and their young children’s hair cortisol concentrations (HCCs)—a physiological marker of cumulative hypothalamic pituitary adrenal (HPA)-axis activity. Using data from 127 parent–child dyads collected by two of six ACF-funded Buffering Toxic Stress consortium sites, we tested interaction effects of parental ACEs with parental EA on young children’s (*M*_*age*_ = 18.38, *SD*_*age*_ = 7.10) HCC. Results revealed curvilinear main effects such that higher parental ACEs were significantly associated with greater HCC and stronger associations occurred at higher levels of parental ACEs. However, this association was moderated by parental EA. Thus, among children with higher parental history of ACEs, children of parents with higher EA had lower HCC compared to children of parents with lower EA. These findings provide support for the risk-buffering and risk-exacerbating role of parent–child relationship quality (e.g., EA) for the transmission of parents’ early life adversity on their children’s HPA-axis activity, documented here in a racially and ethnically diverse sample of children and parents served by Early Head Start. Findings suggest that intervention and prevention efforts targeting stress response in children of mothers with childhood adversity should also support parents in building an emotionally available relationship with their children.

Parental history of adverse childhood experiences (ACEs) are associated with disruptions in children’s stress response (Brand et al., 2010), which may increase children’s future risk for psychopathology (Koss & Gunnar, 2018). Parental ACEs can impair one’s ability to provide sensitive caregiving (Plant et al., 2018) contributing to alterations in children’s hypothalamic pituitary adrenal (HPA)-axis functioning that may underlie the intergenerational transmission of adversity (Koss & Gunnar, 2018). However, in the presence of a supportive social relationship or buffer, the *toxic* effects of a chronic or severe stressor (e.g., unmitigated stress response in the absence of supportive relationships) may become *tolerable* (e.g., stress response buffered by supportive relationships; Shonkoff et al., 2009). The current study tested the association between parental history of abuse, neglect, and family dysfunction, or ACEs, and children’s stress response as indicated by children’s hair cortisol concentration (HCC)—a biomarker of chronic activation of the HPA-axis (Stalder et al., 2017). We also examined the potential buffering or moderating role of parental emotional availability (EA), a characteristic of high-quality parent–child relationships (Biringen et al., 2014), in the association between parental ACEs and children’s HCC. Participants in this study were predominantly American Indian and/or Latinx and all were enrolled in Early Head Start, which particularly aims to address the needs of low-income children and families.

## Parental ACEs and Children’s Stress Response

Research directly examining the putative biological and behavioral pathways linking parental history of adversity and children’s stress physiology are limited. Proposed indirect behavioral pathways point to effects of parental ACEs on parental concurrent psychosocial adversity including parenting stress (Lange et al., 2019) and mental illness (e.g., depression) with downstream negative effects on parenting practices (Pereira et al., 2012; Plant et al., 2018), which in turn, compromise HPA-axis activity in children (Sanchez et al., 2015). Additionally, experiences of early childhood trauma in interaction with concurrent life stressors (e.g., financial and relational difficulties) compromise parental HPA-axis functioning (Young et al., 2019). In turn, impaired parental HPA-axis functioning (e.g., flat diurnal cortisol patterns) may compromise parental affective expressions and is associated with withdrawn, intrusive, or frightening behaviors (reflective of lower EA) during parent–child interactions (Juul et al., 2016; Nyström-Hansen et al., 2019). Such disrupted interaction patterns can heighten children’s stress-induced arousal and contribute to emotional and behavioral dysregulation (Crugnola et al., 2019). Proposed direct biological pathways point to programming effects of prenatal exposure to stress hormones that alter children’s stress physiology in utero and after birth (Coussons-Read, 2013). Moreover, evidence from animal models underscores changes in epigenetic signals through which parental stress associated with socioeconomic disadvantage and early childhood adversity is indirectly transmitted to infants (Scorza et al., 2019). Some evidence suggests that children of parents with greater ACEs display lower baseline salivary cortisol levels, lower baseline respiratory sinus arrhythmia, greater increases in cortisol secretion when faced with acute stressors, and, greater activation of sympathetic nervous system, all indicators of a compromised stress response (Brand et al., 2010; Jovanovic et al., 2011). However, measurement of cortisol in saliva, urine, or blood reflect secretion over short periods of time and produce highly variable profiles of HPA-axis activity depending on several factors (e.g., time of day, specific stressor). For instance, when faced with acute stressors, children of mothers with post-traumatic stress disorder display blunted cortisol reactivity profiles (Danielson et al., 2015). These inconsistent findings necessitate examination of a more consistent biomarker of stress exposure such as HCC (Stalder et al., 2017).

### Adversity and HCC

HCC reflects cumulative cortisol in the hair and has been regarded as a preferable method for understanding chronic HPA-axis activity as it provides a retrospective measure of cortisol secretion accumulated over several months (Stalder et al., 2017). Generally, individuals experiencing chronic adversity display elevated HCC (Stalder et al., 2017). Relatively few studies have examined determinants of HCC in children and have primarily focused on concurrent forms of family adversity mainly in the form of socioeconomic disadvantage (See Gray et al., 2018 for review). In children between 4 and 18 years, elevated HCC is associated with lower parental income and education (Gray et al., 2018; Rippe et al., 2016). Additionally, while some research points to positive links between children’s HCC and trauma exposure, others have found no associations between children’s HCC and other family-level stressors (e.g., intimate-partner violence; Gray et al., 2018). Therefore, it is necessary to examine risk-increasing and risk-protective family-level factors (e.g., caregiving quality) that may account for heterogeneity in associations between children’s HCC and exposure to family-level stressors (e.g., maternal ACEs). Most studies have focused on effects of concurrent socioeconomic adversity (e.g., maternal education, single mother, maternal perceived social support) on child HCC. To date, most studies have neglected family-level stressors such as maternal history of abuse, neglect, and family dysfunction, with two notable exceptions that demonstrated similar findings from majority middle- and high-income samples. Slopen et al. (2018) followed 660 children from birth to 4 years and found greater HCC emerging at 3–4 years (but not at 1 or 2 years) in children of mothers with a greater number of traumatic life events. Additionally, in a sample of 30 newborns, marginally higher dehydroepiandrosterone (i.e., steroid hormone thought to counteract cortisol) was found in the scalp hair of newborns of mothers who had a history of childhood maltreatment (Schury et al., 2017). However, no association was found between maternal childhood trauma and newborns’ HCC (Schury et al., 2017), an important counter to the prenatal programming pathway. Neither of these studies however, examined the role of parent–child relationship quality, leaving a considerable gap in knowledge for the interaction effects of family-level stressors (e.g., parental ACEs) and parent–child relationship quality (e.g., parental EA) on children’s HCC. The current study is the first to examine links between parental ACEs and children’s HCC and the moderating role of parent–child relationship quality, a potential social buffer against negative effects of maternal ACEs on children’s HCC.

### Parental ACEs and Parent–Child Relationship Quality

High-quality parent–child relationships are characterized by a parent’s consistent ability to accurately perceive, interpret, and accept children’s psychological states (Zeegers et al., 2017), and sensitivity or tendency to display appropriate affect and behavioral responsiveness to children’s emotional and behavioral cues (Biringen et al., 2014). Additionally, parental positive emotional expressions, verbal and physical warmth, and supportive regulatory strategies in response to children’s distress are consistent indicators of high-quality parent–child relationships (Morris et al., 2017). EA, one indicator of parent–child relationship quality, is a multidimensional and dyadic construct indicated by the affective and behavioral dimensions (Biringen et al., 2014). Emotionally available parents are optimally attuned and display appropriate affect and responsiveness to their child’s emotional and behavioral cues while less emotionally available parents are characterized as complicated, detached, and/or problematic in their dyadic behavioral and emotional interactions. Although child characteristics also influence the parent–child relationship, its quality is not immune from the negative influences of parents’ own unresolved history of adverse childhood experiences (Kim et al., 2014; Thompson-Booth et al., 2019).

#### Parental ACEs and EA

Except for null findings in one low-risk sample (Sexton et al., 2017), studies have consistently shown that parents with higher ACEs are also at higher risk for disturbances in parent–child relationships including greater parental distress (e.g., depression, partner-conflict) and greater stress associated with their child’s difficult behaviors (Lange et al., 2019). Maternal history of abuse, neglect, and family dysfunction is associated with lower EA (Fuchs et al., 2015; Kluczniok et al., 2016; Pereira et al., 2012), greater hostility and intrusiveness (Bailey et al., 2012; Moehler et al., 2007), and no significant improvements in EA during the first year of their infants’ life (Fuchs et al., 2015), suggestive of disruptions in normative caregiving.

### Parental EA and Children’s Stress Response

Mounting evidence highlights the social buffering role of supportive caregiving behaviors on HPA-axis activity in rodents, and in non-human and human primates (Gunnar et al., 2015). Supportive caregiving facilitates regulation of HPA-axis activity whereas non-supportive and insensitive caregiving contribute to elevated levels of cortisol and disruptions in HPA-axis function (Johnson et al., 2018), including blunted diurnal cortisol profiles (Tarullo & Gunnar, 2006). Although some evidence supports the link between maternal EA and physiological biomarkers of stress response in children, evidence for the buffering role of EA on links between maternal ACEs and children’s stress response is limited. For instance, higher maternal EA is associated with optimal diurnal patterns of salivary cortisol in 1–3 months old infants (Philbrook et al., 2014) and greater change in cortisol secretion in response to maternal separation in toddlers (Sturge-Apple et al., 2012). Additionally, higher maternal intrusiveness, reflective of lower EA, is associated with greater increases in HCC 3 months post kindergarten entry (Rickmeyer et al., 2017). Therefore, we would expect that parental EA would serve to attenuate the effects of parental ACEs on children’s physiological stress response as evidenced by HCC.

### Current Study

Most research to date has examined effects of maternal ACEs on children’s HPA-axis activity utilizing salivary cortisol with inconsistent findings associated with variability of cortisol secretion in short-term measurement occasions. Additionally, studies of determinants of HCC—a retrospective biomarker of cumulative HPA-axis activity—suggest mixed findings on links between children’s HCC and early non-socioeconomic stressors (e.g., trauma exposure) pointing to the need for examination of family-level stressors and protective factors to explain observed heterogeneity in findings controlling for child age. Both Latinx and American Indian children and families in the USA experience considerable mental and physical health disparities perpetuated and exacerbated by contextual, economic, and historic stressors (Parra-Cardona et al., 2017; Sarche et al., 2017). Also, these families experience lower access to and availability of culturally and contextually appropriate evidence-based intervention and prevention programs that promote positive parenting (Parra-Cardona et al., 2017; Walters et al., 2020). Therefore, we examined the buffering role of parental EA in the association between parental ACEs and children’s HCC in a sample of predominantly American Indian and Latinx families whose young children were enrolled in Early Head Start. We hypothesized that parental ACEs would be associated with greater HCC in children. We also hypothesized that greater parental ACEs would be associated with higher HCC for children with less emotionally available parents, but with lower HCC for children with more emotionally available parents.

## Method

### Participants

Data was collected in two out of the six Buffering Toxic Stress Consortium university-Early Head Start (EHS) partnership sites funded by the Administration for Children and Families (Buffering Toxic Stress Consortium Principal Investigators et al., 2013). A total of 127 mother–child dyads enrolled in EHS and living in a semirural Southern plains American Indian community (site 1*, n* = 95) or in an urban metropolitan area (site 2*, n* = 32) participated in the study. Children (58% boys) were between 5 and 44 months at enrollment (*M* = 18.38, *SD* = 7.10). Most parents identified as the target child’s biological mother (95.1%), and less than 5% identified as the target child’s adoptive mother, foster mother, grandmother, or stepfather. Participants identified as exclusively, or a combination of, three major racial categories including American Indian, White, and African American. About 1/3 of the sample identified as ethnically Latinx (Table 1). All data analyzed were baseline data collected prior to families’ participation in each site’s intervention—both of which were aimed at supporting positive parent–child interactions. For site 1, Parent Child Interaction Therapy (Eyberg et al., 1995), a live parent-coaching program, was implemented in an exploratory way to assess feasibility and cultural and contextual fit given prior work to increase its cultural alignment for American Indian communities more broadly (BigFoot & Funderburk, 2011). For site 2, parents were randomly assigned to Filming Interactions to Nurture Development (Fisher, 2012), a video coaching program that emphasized caregivers’ strengths and positive responsiveness to child cues.

**Table 1:** Summary of sample demographics (*N* = 127)

	*n*	*M* (*SD*)	Range
Parental age (years)	125	27.57 (7.43)	16–49
Economic hardship	126	0.75 (1.08)	0–4
Marital status	%		
Single	28.3		
Married	33.9		
Widowed	1.6		
Separated	2.4		
Divorced	8.7		
In relationship living with partner	21.3		
In relationship not living with partner	2.4		
No response	1.6		
Education	%		
< High school	21.3		
High school graduate/GED	22.0		
Associate/Vocational	15.0		
Some college	26.8		
College graduate	8.7		
College +	5.5		
No response	0.8		
Annual income	%		
10 K or lower	11.0		
10 K–24 K	25.2		
25–49 K	22.8		
50 k–74 K	8.7		
75 K +	3.1		
No response	29.1		
Parental race	%	Child race	%
White only	40.9	White only	26.8
Black only	1.6	Black only	0.8
AIAN only	33.1	AIAN only	23.6
White + Black	-	White + Black	0.8
White + AIAN	15.7	White + AIAN	18.9
Black + AIAN	0.8	Black + AIAN	0.8
White + Black + AIAN	1.6	White + Black + AIAN	5.5
Biracial	1.6	Biracial	2.4
No response	4.7	No response	20.5
Parental ethnicity	%	Child ethnicity	%
Latino/a	32.3	Latino/a	27.6
Not Latino/a	66.9	Not Latino/a	25.2
No response	0.8	No response	47.2

AIAN = American Indian Alaska Native

### Measures

#### Parental ACEs

Each site used slightly different versions of ACEs questionnaires to retrospectively assess parental exposure to the same 10 categorical stressors in the first 18 years of life. The same 10 categorical stressors were measured in both sites including emotional, physical, and sexual abuse, emotional and physical neglect, parental divorce/separation, domestic violence against mother, household substance abuse, household mental illness, and incarceration of household member. For site 1, parental ACEs were measured using a 10-item adverse childhood experiences measure (Anda et al., 2009). For site 2, maternal ACEs were measured using the original long form (68 items) of the Family Health History Questionnaire (Felitti et al., 1998). In site 2, multiple items assessing the same stressor were summed to create each of the 10 categorical stressors. Participants responded with a 1 (*yes*) or 0 (*no*) to questions related to each stressor. Responses were dichotomized for each of the 10 stressors and summed to create a total ACE score ranging from 0 indicating *no exposure* to 10 indicating exposure to all the same 10 stressors for both sites. ACEs are regarded reliable and valid for assessing adversity-related risk in population-based and clinical research contexts (Bethell et al., 2017). Cronbach’s alpha for the current sample was 0.81.

#### EA

Quality of the parent–child relationship was assessed via the Emotional Availability System (4th ed., Biringen, 2008). Parental EA was coded observationally using video-taped parent–child interactions detailed in the procedures section below. There are four adult EA Scales (sensitivity, structuring, non-intrusiveness, non-hostility). First, parents were rated on these 4 dimensions from 1 (*low*) to 7 (*high*). Next, EA-Zone scores were rated on a dimensional 1–100 global rating scale for parents as a summary of the EA Scales coded above, as well as to categorize EA into four distinct zones including: Emotionally Available (81–100), Complicated (61–80), Detached (41–60), and Disturbed/Traumatized (1–40). Scores ≤ 80 are considered less emotionally available, while scores ≥ 81 are considered more emotionally available. EA has good psychometric properties with demonstrated reliability and validity in a variety of caregiving contexts over a wide range of child ages (Biringen et al., 2014). Video-taped parent–child interactions were coded by two trained coders certified as reliable by the developer of the EA coding system (Biringen, 2008). Inter-observer agreement of .80 was achieved prior to independent coding for parent EA Scales dimensions and parent EA zones. During independent coding, we maintained agreement of .80 or higher on double-coded cases (greater than 70% of cases) with weekly observer meetings to resolve discrepancies and arrive at final scores for more difficult to code cases. Inter-observer reliability estimates were calculated as intraclass correlations for the direct scores on each EA Scale dimensions (sensitivity .92, structuring .87, non-intrusiveness .74, non-hostility .91), and parent EA Zones (.93). After scoring EA-Zones, the parent scores were dichotomized to create two groups of high EA = 1 (EA-Zone greater than 81) and low EA = 0 (EA-Zone less than 81). These dichotomized scores (% agreement = .78) were used in moderation analyses.

#### HCC

Hair samples were collected from children during center-visits (site 1) or home-visits (site 2). Research assistants were trained in sampling procedures and collected hair from the posterior vertex. Hair samples were taped to foil with the scalp end marked and stored at room temperature until delivery to a lab for analysis. Hair was ground, cortisol was extracted, and then measured by immunoassay (Salimetrics, LLC, State College, PA) according to previously published methods (Hoffman et al., 2017) with average intra and inter-assay coefficients of variation (CV) 2.7% and 13.3%, respectively.

### Procedures

Demographic information, study questionnaires, hair samples, and video-taped parent–child interactions were collected during baseline study visits conducted in a room in the EHS center designed to resemble a home environment (site 1) and at home (site 2). For both sites, parent–child interactions lasted 20–25 min and consisted of semi-structured playtime followed by a brief stress task (separation-reunion). During semi-structured playtime, dyads were presented with age appropriate play materials and instructed to interact as they normally would (15–20 min for site 1; 10–15 min for sites 2). For both sites, stress induction involved a brief (30–120 s) separation period during which the parent was asked to leave the room until called back by the research assistant filming the session. Parental EA scores were coded using semi-structured playtime and the separation-reunion task (same for both sites).

### Data Analytic Plan

To test the hypothesis that values in child HCC were a function of parental ACES, and that parental EA moderated this relationship, we examined main and moderation effects using multiple regression analysis in M*plus* 8.1 (Muthén & Muthén, 1998-2017). Missing data (< 10% of cases) were handled using full information maximum likelihood estimation. Following recommendations for moderation analyses with relatively small sample sizes (Wang & Preacher, 2015), we used a Bayesian estimation approach to increase unbiased parameter estimates and statistical power. Following recommendations that analyses of cumulative ACEs scores be conducted on a dose–response continuum (Bethell et al., 2017) and previous findings that 4 or more ACEs are associated with greater risk for negative health outcomes (Felitti et al., 1998), we tested for goodness of fit to determine if linear relations adequately modeled influences of parental ACEs on children’s HCC. Results of curve estimations suggested that in addition to a linear relationship [*F*(1, 108) = 3.99, *p* < .05, *r*^2^ = .03], the association between parental ACEs and child HCC was curvilinear [*F*(2, 107) = 3.47, *p* < .05, *r*^2^ = .06, *Δr*^2^ = .03]. Thus, linear and quadratic functions were used to test the main effects of maternal ACEs on children’s HCC. Following recommendations (Hayes, 2013) that predictor variables not be centered prior to creating quadratic terms (i.e., expected multicollinearity between linear and quadratic term), we did not center linear or quadratic ACEs. Interaction terms for linear ACEs and quadratic ACEs with EA (low EA = 0, high EA = 1) were defined in M*plus* and included in moderation analyses to test interaction effects. Controlling for child age, simple slope tests were used to probe conditional effects of parental EA in the relationship between ACEs and HCC. To further reduce biased parameter estimates and increase power (i.e., decrease type II error) we tested for the difference in simple slopes as recommended for testing with a dichotomous moderator (Robinson et al., 2013). Following recommendations to consider child sex in variations of HCC (Stalder et al., 2017), we tested the relationship between child sex and study variables. Child sex was not significantly related to any of main study variables nor was it related to child HCC in moderation analyses and was therefore excluded from final models. Given significant relationships between study variables and child age (months) and previous inconsistent findings for links between age and child HCC (Gray et al., 2018), child age was included as a covariate in the final models. Although there were site differences in participants’ race/ethnicity (site 1 predominantly American Indian and non-Latinx; site 2 predominantly White/Latinx), site was not correlated with child HCC after controlling for child age (*r* =  −.08, *p* > .10) and not a significant predictor of HCC (*p* > .10) and was excluded in main analyses. There were no racial/ethnic differences in study variables, except for a slightly lower mean of maternal ACEs for Latina mothers (*M* = 1.33, *SD* = 1.46) compared to Non-Latina mothers (*M* = 2.31, *SD* = 2.61, *p* < .05).

## Results

Results of descriptive statistics and bivariate correlations are reported in Table 2. As expected, greater parental ACEs were associated with higher concentrations of child log HCC (pg/mg; *r* = .19, *p* < .05). Additionally, greater parental ACEs were marginally associated with lower parental EA (*r* = .12, *p* < .10) whereas child age was positively associated with parental EA (*r* = .29, *p* < .01) and negatively associated with HCC (*r* =  −.35, *p* < .01). Natural log transformation was used to normalize HCC values (Skewness = 5.87, *SE* = .22; with 16 extreme outliers or data values that were 3 times the interquartile range) and log transformed HCC scores were used in all analyses. Additionally, we performed four separate *χ*^2^ tests of independence to examine group differences in EA for parents falling into 4 dichotomized groups: (1) 0 ACEs, (2) 1–3 ACEs, (3) 4–5 ACEs, and (4) 6 or more ACEs. Groupings were based on recommendations to use a dose–response analysis approach to the study of ACEs (Bethell et al., 2017; Felitti et al., 1998). The only significant difference in EA was found for group 4, *χ*^2^ (1, *N* = 116) = 6.55, *p* < .05. Parents with 6 or more ACEs had lower means on EA (*M* = 0.07, *SD* = 0.27), than did those with 5 or fewer ACEs (*M* = 0.38, *SD* = 0.48), *t*(114) = 2.33, *p* < .05. Additionally, a *χ*^2^ test of independence suggested significant mean differences in HCC between children with parents rated as high versus low in EA (*χ*^2^ = 140.49 (*N* = 110), *p* < .05).

**Table 2:** Results of study analyses

Descriptives and correlations	*n*	*M* (*SD*)	Range	1	2	3	4
1. Parental ACEs	121	2.00 (2.34)	0–10	–			
2. Parental EA	119	0.34 (0.47)	0–1	−.12^*^	–		
3. Child HCC (pg/mg)	115	3.93 (1.89)	−0.81–9.48	.19^**^	0.03	–	
4. Child age (months)	120	18.38 (7.10)	5–44	0.1	.29^***^	−.35^***^	–
Main and moderated effects	*B*	95% CI	*Β*	95% CI			
Child age (months)	−0.11^**^	[−0.15, −0.06]	−.41^**^	[−0.53, −0.26]			
ACEs linear	−0.33	[−0.69, 0.12]	−0.41	[−0.85, 0.16]			
ACEs quadratic	0.07^**^	[.01, 0.11]	.63^**^	[0.09, 1.07]			
EA	−0.12	[−0.99, 0.90]	−0.03	[−0.24, 0.24]			
ACEs linear X EA	1.05^**^	[0.15, 1.95]	.72^**^	[0.11, 1.33]			
ACEs quadratic X EA	−0.16^**^	[−0.29, −0.02]	−.65^**^	[−1.11, −0.07]			

*ACEs* adverse childhood experiences, *EA* Emotional Availability (1 = high EA, 0 = low EA), *HCC* hair cortisol concentration (pg/mg) is in natural log units

*p < .10, **p < .05, ***p < .01, ****p < .001

**Fig. 1:**
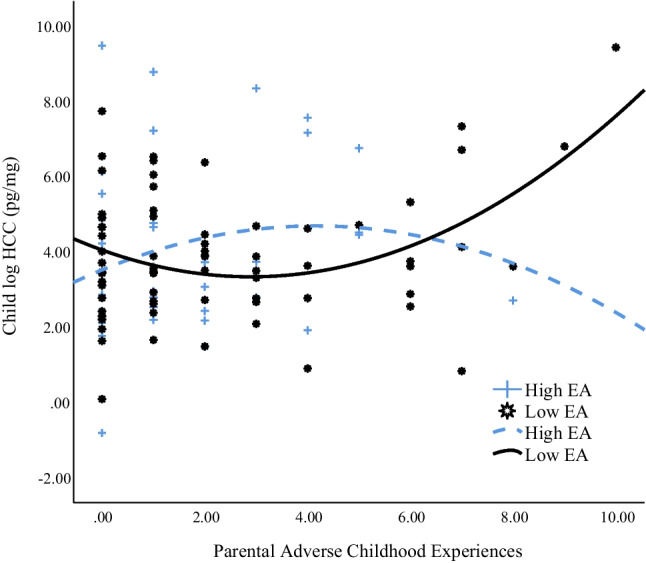
Relationship between parental adverse childhood experiences and child natural log hair cortisol concentrations (pg/mg) as moderated by parental Emotional Availability (EA); High EA R^2^ Quadratic = 0.04, Low EA R^2^ Quadratic = 0.16

Results of main and moderation effects are presented in Table 2 and Fig. 1. Parameter estimates for model fit demonstrated good fit (posterior predictive *p* value = 0.50). The overall model explained 26% of the variance in child HCC. The coefficient of the linear term is the linear component of how HCC changes as parental ACEs change and was not significant in our model (*β* =  −.41, 95% CI [−0.85, −0.16], *p* > .05). The coefficient of the quadratic term (i.e., combined linear and quadratic effects) indicates concavity where effects of ACEs on HCC increase or decrease more at higher or lower values of maternal ACEs (i.e., slope between *x* and *y* becomes more or less positive as *x* changes). Our findings point to a hypothesized positive main effect of the quadratic term (U-shaped) indicating that the slope between ACEs and HCC becomes more positive as ACEs increase (*β* = .63, 95% CI [0.09, 1.07], *p* < .05). This indicates that the association between ACEs and HCC increased (i.e., became more positive) for children of mothers with greater number of ACEs relative to children of mothers with fewer ACEs. Although effects were in the expected direction, we did not find a significant main effect of parental EA on child HCC (*β* =  −0.03, 95% CI [−0.24, 0.24], *p* > .05); however, there was a significant interaction effect between parental ACEs and parental EA as evidenced by the significant *p* values associated with the quadratic interaction term (*β* =  −0.65, 95% CI [−1.11, −0.07], *p* < .05) and the slope difference test (*B* =  −0.16, 95% CI [−0.29, −0.02],* p* < .05).

Probing for interaction effects, the simple slope tests revealed that as hypothesized for children of parents with lower EA, effects of ACEs on HCC increased (e.g., *x*–*y* slope became more positive) as number of ACEs increased (*B* = 0.07, 95% CI [0.01, 0.11],* p* < .05). However, for children of parents with higher EA, the simple slope tests revealed that the hypothesized effects of ACEs on HCC decreased (e.g., *x*–*y* slope became more negative) as number of ACEs increased (*B* =  −0.09, 95% CI [−0.21, 0.01],* p* < .05). Results of the simple slopes suggested that the curvilinear association between parental ACEs and child HCC shifts from a hypothesized positive simple slope coefficient (U-shaped) for children of parents with lower EA to a hypothesized negative simple slope coefficient (inverted U-shaped) for children of parents with higher EA.

For children of parents with lower EA, there was a sharper increase (greater disadvantage) in the positive slope between ACEs and HCC when moving from lower to higher ACEs (e.g., from 4–5 ACEs to 9–10 ACEs; Fig. 1). As the number of ACEs increase, there is a greater disadvantage (i.e., elevated HCC) of being a child of a lower emotionally available parent. However, for children of parents with higher EA, there is a sharper decrease (greater advantage) in the inverse association between parental ACEs and child HCC when moving from lower to higher ACEs (e.g., from 2–5 ACEs to 6–10 ACEs). As the number of ACEs increase, there is a greater advantage of being a child of a *higher* emotionally available parent. Accounting for effects of child age, parental linear and quadratic ACEs, children with higher emotionally available parents had lower HCC (*M* = 3.88, *SD* = 0.38) compared to children with lower emotionally available parents (*M* = 3.96, *SD* = 0.23, *g* = 0.29, 95% CI [−0.11, 0.69]). Hedges’ *g* of 0.29 indicates that the two groups differed by 0.29 standard deviations suggestive of a small to medium effect size^1^.

## Discussion

This is the first study that examined the moderating role of parental EA on the association between parental ACEs and children’s HCC. As hypothesized, we found a positive association between the number of parental ACEs and children’s HCC. Specifically, we found evidence for curvilinear main effects such that as parental ACEs increased, so too did children’s HCC, with sharper increases for children with greater maternal ACEs. These results are consistent with previous findings pointing to links between maternal trauma history and children’s elevated HCC and similarly support a dose–response analysis approach to the study of ACEs (Slopen et al., 2018). Adding to the extant literature, we found that the relationship between parental ACEs and children’s HCC was moderated by parental higher versus lower EA. Specifically, children of mothers with higher EA appeared to be buffered from the effects of their parents’ childhood experiences of abuse, neglect, or family dysfunction. Additionally, the significant curvilinear interactions suggest that the buffering influence of higher parental EA is more advantageous for children of parents with a greater number of ACEs relative to children of parents with fewer ACEs. Conversely, children of parents with lower EA were not buffered from the effects of their parents’ childhood abuse, neglect, and family dysfunction as indicated by children’s elevated HCC. In fact, in the absence of emotionally available caregiving (e.g., positive affect, attentiveness, responsiveness), parental ACEs are an especially robust predictor of children’s elevated HCC. Shonkoff et al. (2009) argued that early life’s unbuffered and chronic stressors disrupt one’s stress response and create vulnerabilities for disease in adulthood. Our findings build on extant literature that support Shonkoff et al.’s model by suggesting that targeting social buffers in early life may contribute to greater shifts from toxic stress to tolerable stress (e.g., indexed by lower HCC) for those facing the greatest adversity (e.g., children of mothers with highest ACEs).

Parents who can be emotionally available despite a history of adverse childhood experiences are likely to have acquired effective coping strategies that are modeled and used to scaffold positive regulatory strategies for their children. In turn, children of parents with greater regulatory strategies are better able to regulate their own stress response (Pat‐Horenczyk et al., 2015). Additionally, the current findings suggest that parental trauma history may need to be addressed to prevent disruptions in caregiving quality and continuity of intergenerational adversity. Parental unresolved trauma has been linked with blunted amygdala response to infant distress (Kim et al., 2014) as well as disrupted processing of infant facial cues (Thompson-Booth et al., 2019). Thus, parents with highly unresolved trauma may benefit from interventions targeting their trauma-related symptoms such as ability to self-regulate, in tandem with direct support of affective and behavioral responsiveness and acceptance of child’s emotional and behavioral bids. Accordingly, accumulating neuroscience evidence suggests that moving from attachment disorganization to organization and from unresolved trauma to resolved is instrumental in preventing the intergenerational transmission of adversity (Iyengar et al., 2019). Our results especially highlight supporting an emotionally available parent–child relationship as a protective mechanism among children whose parents have a history of childhood abuse, neglect, and family dysfunction, particularly as parental ACEs increase. In terms of implications for prevention science, our findings suggest targeting positive caregiving—for example, through the implementation of culturally and contextually aligned evidence-based parenting programs—is important for mitigating the effect of parental ACEs on children’s stress response in Latinx and American Indian communities.

### Limitations and Future Directions

The current study does not allow for examination of the mediational mechanisms through which parental ACEs exert an influence on a physiological marker of children’s stress regulation. In our sample, roughly 10% of mothers were pregnant with the target child before age 18, so a small number of parental ACEs may have occurred during pregnancy and may explain variation in child HCC as resulting from prenatal stress exposure. Additionally, the extant literature suggests that individuals who experience early childhood trauma may carry the sequelae of those experiences forward into their adult life in a variety of ways that can influence children’s development—including through impairments in HPA-axis functioning with downstream effects on parental mental health, parenting stress, as well as financial and relational instability (Juul et al., 2016; Plant et al., 2018)—all of which may need to be addressed before promotion of EA is feasible. Additionally, less is known about the mediational role of adult stress exposure and conditional effects on parenting quality and HCC. For instance, it is possible that adult life stressors (not examined here) commonly associated with history of ACEs (e.g., parenting stress, perceived support, relational and financial difficulties) were lower for our high EA mothers than our low EA mothers. In fact, in our sample current parental depression and anxiety were associated with higher ACEs, but not with parental EA or child HCC. However, more economic hardship (e.g., difficulty paying bills) and lower income categories were associated with lower EA but not with ACEs nor with child HCC. Thus, future work must examine the role of maternal concurrent life stress, psychological distress (e.g., depression), and physiological markers of stress in tandem with history of ACEs to disentangle effects on parenting quality and child HCC and inform promotion efficacy of EA for parents with unresolved trauma. Additionally, both elevated and dampened HCC levels may be associated with experiences of adversity (Khoury et al., 2019); however, age-related variations are thought to partly explain these findings. Overall, previous results related to children’s age and HCC have been mixed (Gray et al., 2018). In the current cross-sectional sample, older children had lower HCC while in Slopen et al.’s longitudinal study (2018), greater HCC emerged for 3 and 4 years (but not at 1 or 2 years) consistent with mixed findings on age and HCC. Additionally, some findings suggest that adversity related HCC levels are dampened in middle childhood to adolescence (Gray et al., 2018). Therefore, future studies must examine HCC in longitudinal samples accounting for interactions between time-related effects and family-level stressors. Finally, our findings are limited by lack of behavioral markers of child stress, lack of other child and parenting factors that contribute to HCC (e.g., child’s own trauma history, behavior problems, maternal regulatory strategies), and not generalizable across all groups of underserved communities with unique experiences of culturally and contextually salient stressors (e.g., African-Americans).

### Conclusion

Parental history of childhood abuse, neglect and family dysfunction is associated with disruptions in children’s HPA-axis activity, specifically elevated HCC. However, this association is moderated by parental EA underscoring the role of high-quality parent–child relationship in buffering children’s HPA-axis activity from putative direct and indirect effects of parental ACEs. As parents with histories of early adversity transition into parenting, additional prevention support may be warranted to prevent disruptions in caregiving quality. Thus, prevention and intervention programs targeting children’s HPA-axis activity should support parents with a history of abuse, neglect, and family dysfunction and promote EA. Promotion of EA for parent–child dyads may in turn reduce the intergenerational transmission of adversity.

## Footnotes

1 Hedges’ *g* is a measure of weighted effect size used for unequal sample sizes (37 emotionally available vs. 73 not emotionally available).
