# Correction to: Emotional Availability as a Moderator of Stress for Young Children and Parents in Two Diverse Early Head Start Samples

**DOI:** 10.1007/s11121-024-01751-1

**Published:** 2024-11-21

**Authors:** Neda Senehi, Marjo Flykt, Zeynep Biringen, Mark L. Laudenslager, Sarah Enos Watamura, Brady A. Garrett, Terrence K. Kominsky, Hannah E. Wurster, Michelle Sarche

**Affiliations:** 1 Department of Psychiatry, University of Colorado Anschutz Medical Campus, 31001 E 17th Place, Aurora, CO USA; 2 Faculty of Social Sciences/Psychology, Tampere University, Tampere, Finland; 3 Faculty of Medicine/Psychology, University of Helsinki, Helsinki, Finland; 4 Department of Human Development and Family Services, Colorado State University, Fort Collins, USA; 5 Department of Psychology, University of Denver, Denver, USA; 6 Cherokee Nation Behavioral Health, Tahlequah, OK USA; 7 Colorado School of Public Health, University of Colorado Anschutz Medical Campus, Aurora, USA

**Correction to: Prevention Science**

10.1007/s11121-021-01307-7

The article "Emotional Availability as a Moderator of Stress for Young Children and Parents in Two Diverse Early Head Start Samples", written by Neda Senehi, Marjo Flykt, Zeynep Biringen, Mark L. Laudenslager, Sarah Enos Watamura, Brady A. Garrett, Terrence K. Kominsky, Hannah E. Wurster, and Michelle Sarche, was originally published electronically on the publisher’s internet portal on 13 November 2021 without open access. With the author(s)’ decision to opt for Open Choice the copyright of the article changed on 04 November 2024 to © The Authors 2024 and the article is forthwith distributed under a Creative Commons Attribution 4.0 International License, which permits use, sharing, adaptation, distribution and reproduction in any medium or format, as long as you give appropriate credit to the original author(s) and the source, provide a link to the Creative Commons licence, and indicate if changes were made. The images or other third party material in this article are included in the article’s Creative Commons licence, unless indicated otherwise in a credit line to the material. If material is not included in the article’s Creative Commons licence and your intended use is not permitted by statutory regulation or exceeds the permitted use, you will need to obtain permission directly from the copyright holder. To view a copy of this licence, visit http://creativecommons.org/licenses/by/4.0/.

The original article has been corrected.

